# Medication-induced lung disease in children

**DOI:** 10.1007/s00247-025-06420-1

**Published:** 2025-11-25

**Authors:** Andrew H. Schapiro, Kristen L. Ruff, R. Paul Guillerman

**Affiliations:** 1 Department of Radiology, Cincinnati Children’s Hospital Medical Center, 3333 Burnet Avenue, Cincinnati, OH 45229 USA; 2 University of Cincinnati College of Medicine, Cincinnati, United States

**Keywords:** Children, Computed tomography, Drug-induced, Lung disease, Medication-induced

## Abstract

Medication-induced lung disease is rare in children but can be associated with considerable morbidity and mortality and can alter treatment regimens for children with a variety of conditions. Medication-induced lung disease tends to occur in association with certain categories of medication and tends to manifest as one of several patterns of lung disease on CT. We review classes of medications associated with medication-induced lung disease, clinical diagnosis and management of the condition including the role of the radiologist, and CT patterns of disease to enable the radiologist to more fully contribute to multidisciplinary diagnosis and potentially be the first to recognize and suggest the possibility of this condition.

**Graphical Abstract:**

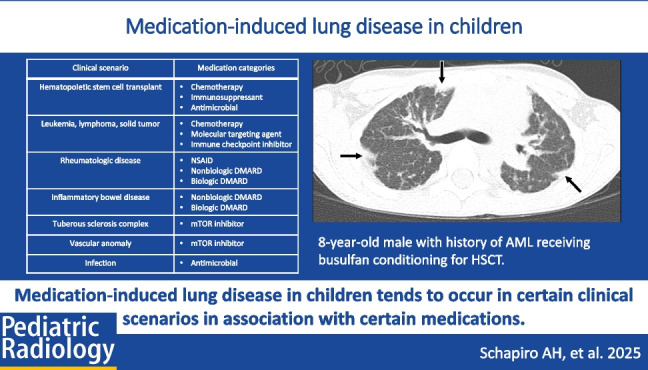

## Introduction

Medication-induced lung disease is rare in children but has been recognized in studies of diffuse lung disease in children ranging from less than 2 years of age to 18 years of age as well as in a number of case series and case reports [1, 2]. The pediatric radiologist may be the first to suggest medication-induced lung disease, potentially prompting change in therapy that can spare a child morbidity and even mortality. However, in our experience, medication-induced lung disease is often not considered in the differential diagnosis of lung disease on imaging, likely due to lack of recognition and relative rarity. It is our goal to familiarize the pediatric radiologist with this entity to prompt consideration in the correct clinical setting and hopefully facilitate earlier clinical diagnosis and treatment.

We will first review classes of medications associated with medication-induced lung disease in children, discuss the path to diagnosis, and briefly describe management. We will then discuss the role of the radiologist in diagnosis and management before describing patterns of medication-induced lung disease in children encountered on computed tomography.

## Medications

Numerous medications are reported to be associated with medication-induced lung disease, and an exhaustive list is beyond the scope of this article. However, consideration of categories of medications associated with medication-induced lung disease and the clinical scenarios in which these categories of medications tend to be used can help the pediatric radiologist identify when to have a higher suspicion for medication-induced lung disease (Table 1) [3]. Categories of medications associated with medication-induced lung disease include chemotherapy agents, immune checkpoint inhibitors, molecular targeting agents, disease-modifying anti-rheumatic drugs (DMARDs), arrhythmia medications, and antibiotics.

**Table 1:** Common clinical scenarios in which medication-induced lung disease should be considered

Clinical scenario	Medication categories	Differential diagnosis	Important points
Hematopoietic stem cell transplant (HSCT)	• Chemotherapy • Immunosuppressant • Antimicrobial	• Infection • Edema • Transfusion-related acute lung injury (TRALI) • Idiopathic pneumonia syndrome • Diffuse alveolar hemorrhage • Peri-engraftment respiratory distress syndrome • Pulmonary veno-occlusive disease (PVOD) • Graft-versus-host disease (GVHD)	• While medication-induced lung disease can occur at any stage following HSCT, other pulmonary complications follow a predictable timeline after HSCT, so knowing the timing since HSCT is important • TRALI occurs within 6 hours of administration of blood or blood products • PVOD appears as centrilobular ground-glass opacities, interlobular septal thickening, and lymphadenopathy in addition to findings of pulmonary hypertension • GVHD is more of a concern in patients with allogeneic HSCT than autologous HSCT
Leukemia, lymphoma, solid tumor	• Chemotherapy • Molecular targeting agent • Immune checkpoint inhibitor	• Infection • Edema • Radiation pneumonitis • Pulmonary embolism (PE)-related hemorrhage/infarction • Tumor spread • Leukemic infiltration • Leukostasis • Leukemic cell lysis pneumopathy • Hyperleukocytic reaction	• Radiation pneumonitis tends to be confined to the radiation field • PE-related hemorrhage/infarction tends to manifest as peripheral wedge-shaped foci of consolidation • Leukemic infiltration tends to occur at the terminal stage of disease • Leukostasis and hyperleukocytic reaction tend to occur in AML patients with high WBC count (generally >100,000/mm^3^) • Leukemic cell lysis pneumopathy occurs within 10–48 hours of chemotherapy initiation in patients with high WBC count (generally >200,000/mm^3^)
Rheumatologic disease	• NSAID • Nonbiologic DMARD • Biologic DMARD	• Infection • Connective tissue disease (CTD)-associated lung disease	• NSIP, OP, and DAD can be seen with both CTD-associated lung disease and medication-induced lung disease, so it is important to establish a temporal relationship with medication administration • Symmetric bilateral subpleural anterior upper lobe involvement along with lower lobe involvement is a disease distribution seen in some CTD-associated lung disease (particularly NSIP and sJIA-associated lung disease) that is not typical of other lung diseases
Inflammatory bowel disease (IBD)	• Nonbiologic DMARD • Biologic DMARD	• Infection • IBD-associated lung disease	• IBD-associated lung disease is rare and most commonly involves the airways, although parenchymal disease (usually organizing pneumonia) can occur
Tuberous sclerosis complex	• mTOR inhibitor	• Infection • Lymphangioleiomyomatosis (LAM) • Multifocal micronodular pneumocyte hyperplasia (MMPH)	• MMPH and LAM present as randomly distributed tiny nodules and cysts, respectively, and can co-exist, both atypical findings for medication-induced lung disease
Vascular anomaly	• mTOR inhibitor	• Infection • Vascular anomaly lung involvement (particularly lymphatic malformation)	• Pulmonary involvement by a lymphatic malformation tends to manifest as thickening of the peribronchovascular and interlobular septal interstitium as well as pleural effusion +/- splenic lesions, bone lesions, and mediastinal involvement • Pulmonary involvement by vascular malformation is more likely to remain stable or slowly progress while medication-induced lung disease is more likely to evolve/migrate
Infection	• Antimicrobial	• Aspiration • Exposure-related lung disease in outpatients (e.g., hypersensitivity pneumonitis, EVALI, smoking-induced acute eosinophilic pneumonia)	• Dependent ill-defined centrilobular/tree-in-bud nodules suggests aspiration or infection rather than medication-induced lung disease • Exposure history important for eliminating exposures as a possibility

*NSAID,* nonsteroidal anti-inflammatory drug; *DMARD,* disease modifying anti-rheumatic drug; *mTOR* mammalian/mechanistic target of rapamycin; *EVALI,* electronic cigarette or vaping product use-associated lung injury; *AML,* acute myeloid leukemia; *WBC,* white blood cell; *NSIP,* nonspecific interstitial pneumonia; *OP,* organizing pneumonia; *DAD,* diffuse alveolar damage

**Fig. 1:**
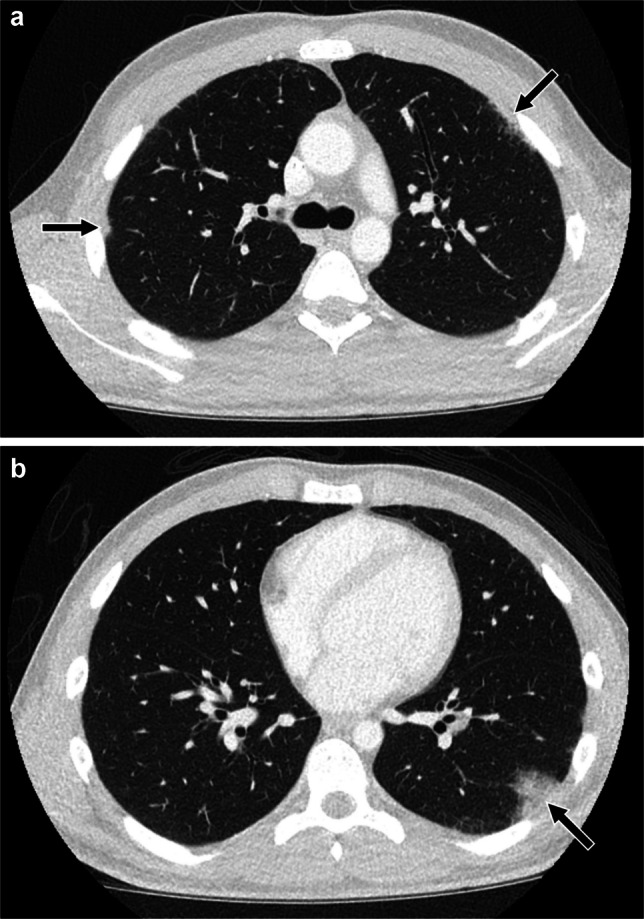
A 17-year-old male who developed exercise intolerance and progressive decline in diffusion capacity on pulmonary function testing during the course of several cycles of chemotherapy that included bleomycin for testicular mixed germ cell tumor. He smoked marijuana, but this was not a new or changed habit, and his family members smoked cigarettes outside the home. There were no clinical signs or symptoms of infection, and his metastatic disease had been responding to treatment. **a-b** Axial CT images with contrast showing multifocal bilateral peripheral consolidation (*black arrows*) that could be seen with organizing pneumonia or chronic eosinophilic pneumonia, with lack of peripheral eosinophilia at the time favoring the former. No pulmonary embolus was seen. Clinical and imaging findings were thought to likely relate to bleomycin-induced lung disease, but bleomycin was continued given that the patient only had one more cycle left and his diffusion capacity had not reached the threshold for bleomycin dose adjustment according to protocol. His symptoms and lung imaging findings had resolved 6 months later, after chemotherapy had been completed, and his pulmonary function testing had substantially improved supporting a diagnosis of bleomycin-induced lung disease

**Fig. 2:**
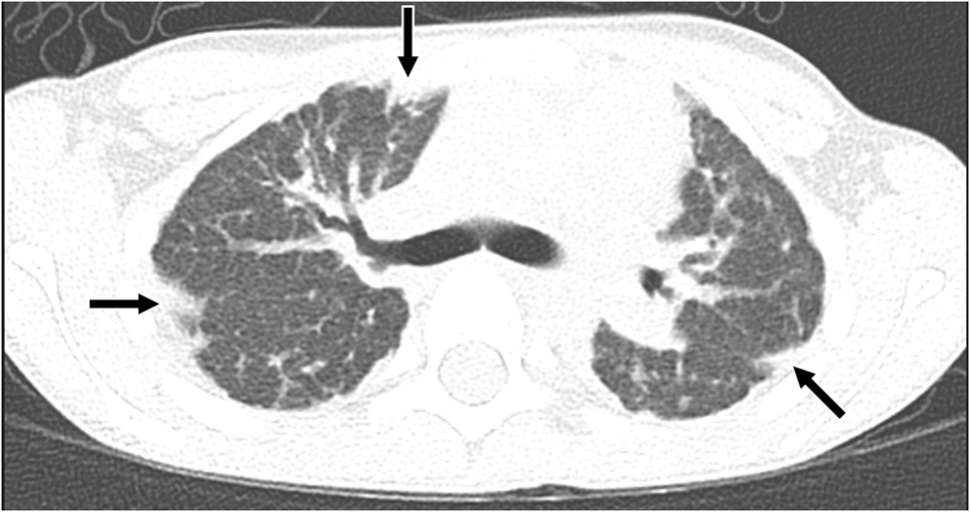
An 8-year-old male with history of acute myeloid leukemia receiving busulfan conditioning for hematopoietic stem cell transplant. Axial CT image without contrast shows multifocal bilateral areas of subpleural consolidation (*black arrow*s). Lung biopsy revealed pleural, subpleural interlobular septal, and patchy alveolar fibrosis with endogenous lipopneumonia and obliterated bronchioles. Temporal relationship of lung disease to administration of a medication known to cause lung disease and lack of alternative explanation supported a diagnosis of busulfan-induced lung disease

Chemotherapy agents and immune checkpoint inhibitors are most often prescribed in hematology and oncology patients, although some may also be used in solid organ and hematopoietic stem cell transplant patients as well as in rheumatology patients. Among chemotherapy agents, the alkylating agent bleomycin is particularly associated with medication-induced lung disease [4–6] (Fig. 1). However, multiple other alkylating agents have been reported to cause medication-induced lung disease in children including cyclophosphamide, nitrosoureas such as carmustine and lomustine, busulfan, and melphalan [6–8] (Fig. 2). Antimetabolite agents such as methotrexate and cytarabine (ARA-C), tubulin agents such as vincristine, podophyllotoxins such as etoposide, and topoisomerase inhibitors such as irinotecan are also associated with lung toxicity [9–13]. Immune checkpoint inhibitors targeting the programmed cell death protein (PD-1), programmed cell death ligand 1 (PD-L1), or cytotoxic T-lymphocyte antigen 4 (CTLA-4) cell surface receptors to modulate T-cell activity against tumor cells have been linked with medication-induced lung disease in adults, and reports of lung disease in children and young adults are emerging, particularly in association with the PD-L1 agent pembrolizumab [14, 15].

**Fig. 3:**
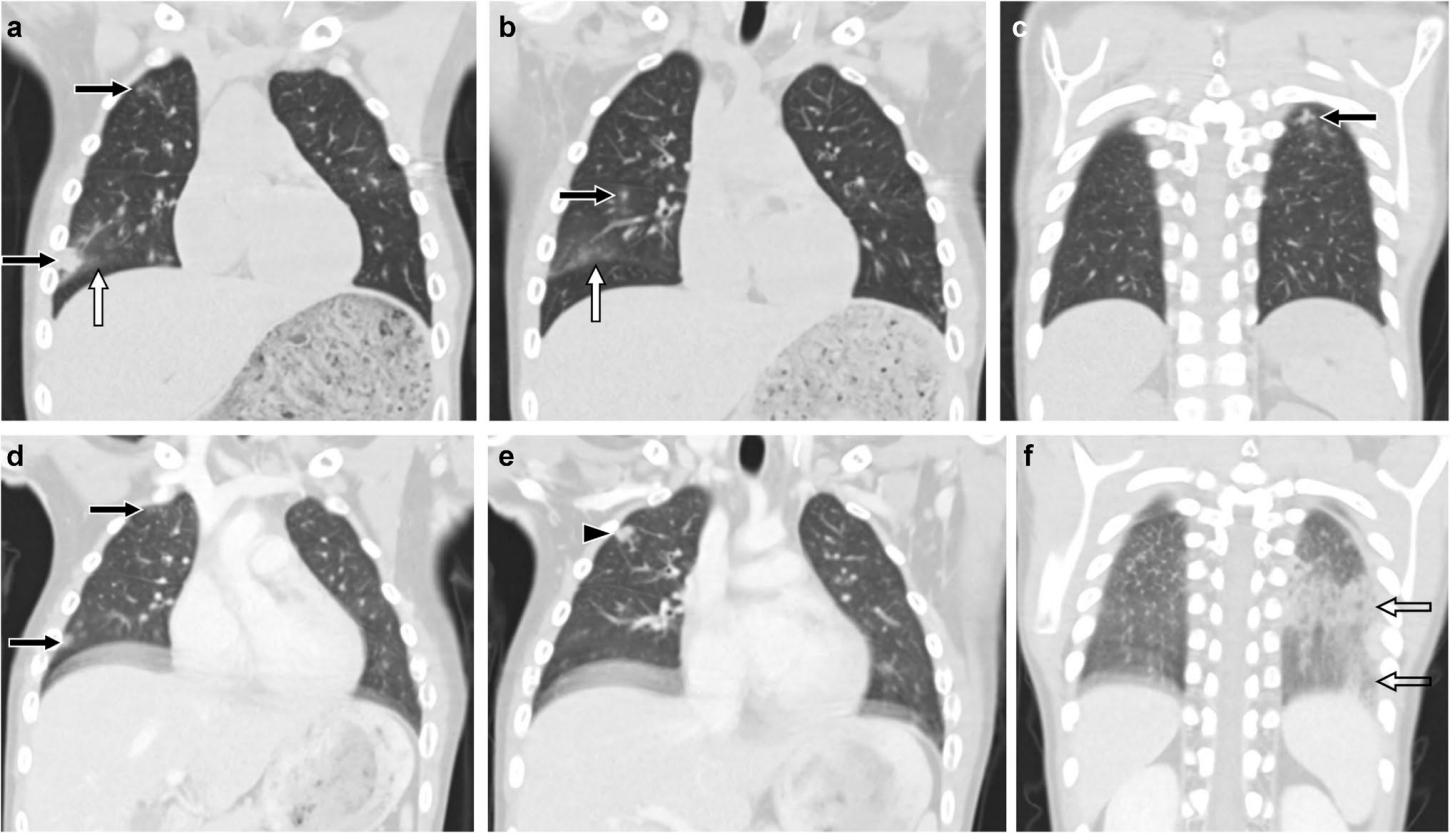
A 10-year-old male receiving sirolimus for tuberous sclerosis complex who presented with fever, achiness, elevated inflammatory markers, and multifocal opacities on chest radiograph in the setting of an elevated sirolimus level of >60 ng/dl (above maximum limit of detection; target ~30 ng/dl for this patient). **a-c** Coronal CT images without contrast obtained at the time of admission showing multiple bilateral ill-defined nodules (*black arrows*) and ground-glass opacity (*white arrow*). **d-f** Coronal CT images with contrast obtained 8 days later showing decreased size of previously seen nodules (*black arrows*) and development of new nodules in the right upper lobe (*arrowhead*) as well as consolidation and ground-glass opacity in the left lower lobe (*open arrow*) (i.e., waxing and waning lung findings). Some of the nodules also remained unchanged in size (not shown). Extensive work-up for infectious, oncologic, and rheumatologic etiologies was unrevealing, fever resolved with withholding of sirolimus and initiation of steroids, and chest CT findings had resolved on follow-up imaging obtained approximately 7 weeks later supporting a diagnosis of sirolimus-induced lung disease

Molecular targeting agents that can be associated with medication-induced lung disease in children include mammalian target of rapamycin (mTOR) inhibitors such as sirolimus and monoclonal antibodies such as the anti-CD20 monoclonal antibody rituximab and the CD30 targeting agent brentuximab vedotin [16–21] (Fig. 3). This category of agents may be utilized in a variety of clinical settings, including in the management of patients with tuberous sclerosis complex, vascular anomalies, hematologic conditions, and following organ transplantation.

DMARDs may be encountered in gastroenterology patients with inflammatory bowel disease, rheumatology patients, and transplant patients. DMARDs associated with medication-induced lung disease include both nonbiologic agents such as methotrexate and azathioprine, and biologic agents such as interleukin inhibitors [6, 22–26].

**Fig. 4:**
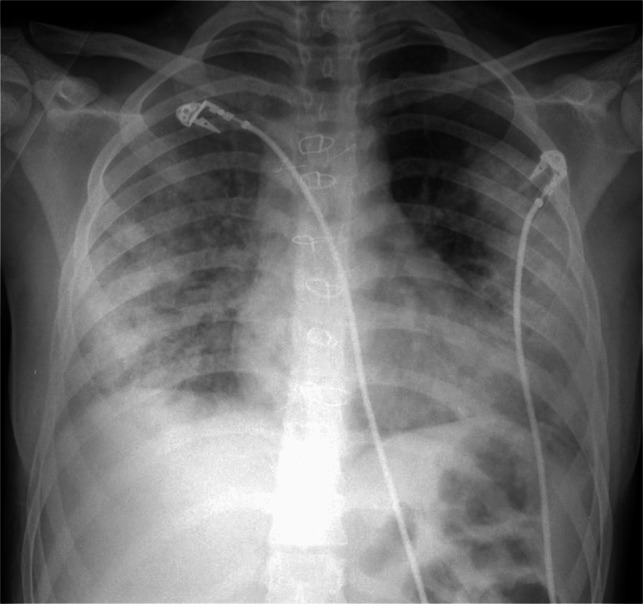
A 17-year-old female with history of cystic fibrosis and lung transplant receiving doxycycline. Anteroposterior chest radiograph showing extensive bilateral peripheral predominant consolidation typical of chronic eosinophilic pneumonia. Temporal relationship to doxycycline administration and response to steroid therapy supported a diagnosis of doxycycline-induced lung disease

Medication-induced lung disease associated with antibiotic use may be seen in children receiving antibiotics for either treatment or prophylaxis of infection, including trimethoprim-sulfamethoxazole, nitrofurantoin, and tetracyclines like doxycycline and minocycline [27–30] (Fig. 4).

Reports of antiarrhythmic agent-associated lung disease in children predominantly involve amiodarone in infants [31–33].

## Clinical diagnosis and management

Establishing the diagnosis of medication-induced lung disease is a challenge, requiring a high index of suspicion, a thorough diagnostic evaluation, and multidisciplinary collaboration. Key to the diagnosis is the identification of a temporal relationship of lung disease to medication administration and exclusion of other reasonable competing diagnoses. However, establishing a temporal relationship between medication administration and lung disease can be difficult given that the length of time between medication initiation and development of lung disease is unpredictable, with presentation potentially acute, subacute, or sometimes delayed for years [34]. For instance, pulmonary fibrosis can progress and become symptomatic and even fatal many years after carmustine exposure, and life-threatening pneumonitis can be precipitated by supplemental oxygen years after bleomycin use [8, 35]. Improvement of lung disease after medication removal or recurrence after re-challenge provides further evidence of medication-induced lung disease, but the associated lung injury does not always improve following drug removal and may even continue to progress [34, 36].

Clinical symptoms and signs, laboratory testing, ancillary testing such as bronchoscopy with bronchoalveolar lavage (BAL) and pulmonary function testing (PFT), imaging findings, and even histopathology of medication-induced lung disease tend to be nonspecific with regard to etiology and are at best supportive of the working diagnosis of medication-induced lung disease. Clinical symptoms, when present, tend to include cough, shortness of breath, and/or fever, with lung auscultation tending to demonstrate crackles, if anything. Laboratory testing is generally most helpful for identifying alternative causes of lung disease but may demonstrate eosinophilia in some cases of medication-induced lung disease manifesting as eosinophilic pneumonia or hypersensitivity pneumonitis [37]. BAL is often most helpful for excluding infection, an important differential diagnosis consideration, but eosinophilia or lymphocytosis with CD-8 cell predominance can be seen [38]. Pulmonary function tests tend to demonstrate a restrictive pattern and decreased DLCO values, findings seen with other interstitial lung diseases [39]. Imaging findings tend to be bilateral, but in our experience, it is not uncommon for findings to be asymmetric or even unilateral [34]. Biopsy may be performed when the etiology of lung disease remains unclear, and entities with different treatment strategies remain in the differential diagnosis. Biopsy can help exclude recurrent malignancy in oncology patients and infection and can identify the pattern of lung injury, but is unlikely to confirm the diagnosis of medication-induced lung injury given that the histological patterns seen in medication-induced lung disease can be due to other etiologies [34].

Given that features of medication-induced lung disease tend to be nonspecific, it is important to exclude other differential diagnoses before establishing a presumptive diagnosis of medication-induced lung disease. Differential diagnosis possibilities to consider include infection (including pneumocystis, cytomegalovirus, fungal, or mycobacterial infection in immunocompromised patients), edema, hemorrhage, lymphoproliferative disease, disease progression (whether that be progression of malignancy or connective tissue disease-associated lung disease), tumor pseudoprogression that can be seen in the setting of immune checkpoint inhibitor therapy, radiation pneumonitis, hypersensitivity pneumonitis, and other causes of lung injury not associated with infection or medication use such as transfusion-related acute lung injury (TRALI) [40–42].

Ultimately, establishing a presumptive diagnosis of medication-induced lung disease is challenging and best accomplished through multidisciplinary discussion between radiologists, clinicians, and pathologists.

In asymptomatic patients with suspected medication-induced lung disease based on imaging findings, the suspected culprit medication may sometimes be continued while the patient is monitored if that medication is vital to patient treatment. After treatment, monitoring plays a role in the long-term surveillance of pediatric cancer survivors, given that the cumulative incidence of treatment-related pulmonary complications in pediatric cancer survivors can increase for many years after diagnosis and is a leading cause of late mortality within this population [36, 43, 44].

In symptomatic patients with suspected medication-induced lung disease, the mainstay of treatment is the discontinuation of the suspected offending medication. In patients with more severe medication-induced lung disease, steroids may be administered. When cases remain refractory to medication removal and steroid administration, the use of other medications such as nintedanib has been reported [45].

## Role of the radiologist

**Fig. 5:**
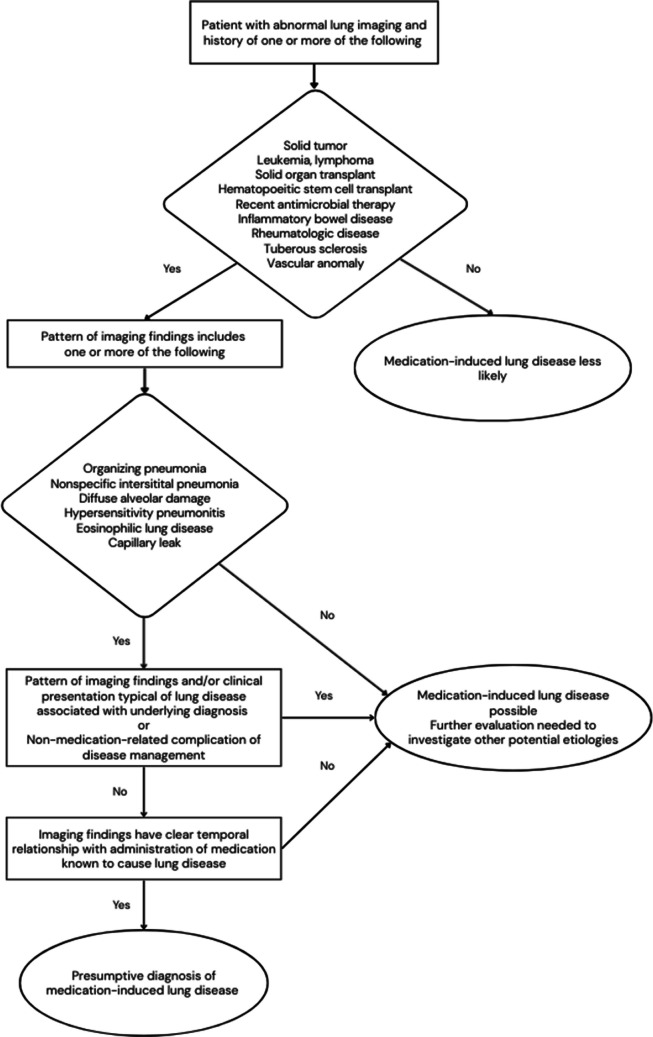
Flowchart illustrating a radiologist approach to medication-induced lung disease in children

Given that clinical findings are typically nonspecific, it is important for radiologists to maintain a high index of suspicion for medication-induced lung disease, recognize lung imaging findings that can be seen with medication-induced lung disease, and suggest the possibility of the diagnosis when such imaging findings are present in the appropriate clinical setting (Fig. 5). On the other hand, it is also important for radiologists to identify when findings suggest that an alternative diagnosis is more likely, as in the case of tree-in-bud nodules that are more suggestive of infection or aspiration [3].

In general, medication-induced lung injury should be considered when there are lung findings on imaging in a patient on a medication known or suspected to cause medication-induced lung disease, as well as in any patient receiving medication who has persistent lung findings and a negative work-up for other etiologies. To identify potential culprit medications, a review of the patient’s medication list is vital. While familiarity with medications more commonly associated with medication-induced lung disease can help facilitate the rapid identification of such medications, publicly available online resources such as pneumotox.com are also helpful for identifying medications associated with medication-induced lung disease, particularly those less commonly associated with medication-induced lung disease, as well as for identifying patterns of lung disease associated with those medications.

In addition to suggesting the possibility of medication-induced lung disease, it is important for the radiologist to characterize the extent and pattern of lung disease, which can correlate with disease severity [46, 47]. However, it should be acknowledged that the imaging pattern may only equate with the histologic diagnosis in approximately half of the cases [48]. Localizing areas of greatest disease involvement may also be helpful for directing bronchoscopy and/or biopsy. After a presumptive diagnosis of medication-induced lung disease is established and management initiated, the radiologist can help further direct management by evaluating changes in lung disease [46, 49].

## Patterns of disease on CT

Potentially recognizable patterns of medication-induced lung disease on CT include organizing pneumonia (OP), diffuse alveolar damage (DAD), nonspecific interstitial pneumonia (NSIP), hypersensitivity pneumonitis (HP), eosinophilic lung disease, and capillary leak (Table 2) [3, 34]. Although the imaging appearance may not clearly match one of the above patterns, medication-induced lung disease should still be considered when nonspecific imaging findings are present in the appropriate clinical setting. More than one of these patterns can be present concurrently, in which case the dominant pattern should be reported [34]. Regardless of whether or not imaging findings can be best categorized as one of the above patterns, it is important to note whether fibrosis is present, typically manifesting as reticulation, architectural distortion, and traction bronchiectasis/bronchiolectasis [50].

**Table 2:** Recognizable patterns of medication-induced lung disease on CT

Patterns of disease	CT features	Imaging differential diagnosis
Organizing pneumonia	• Peribronchovascular and/or peripheral consolidation • Perilobular opacities • Reversed halo/atoll sign • Peribronchial/peribronchiolar nodules with ill-defined margins • Can be migratory	• Infection • Connective tissue disease-associated lung disease • Vasculitis • Inhalation injury (e.g., EVALI, other toxic inhalation, aspiration) • Radiation pneumonitis • Noninfectious sequelae of transplantation (e.g., GVHD, PTLD) • Eosinophilic pneumonia
Nonspecific interstitial pneumonia	• Ground-glass opacity • Reticulation • Traction bronchiectasis/bronchiolectasis • Lower lobe predominant peribronchovascular and/or peripheral distribution	• Connective tissue disease-associated lung disease • Surfactant dysfunction mutation (e.g., SFTPC) • COPA syndrome • SAVI
Diffuse alveolar damage	• Ground-glass opacity • Dependent consolidation • Lung volume loss • Reticulation and traction bronchiectasis/bronchiolectasis with evolution	• Infection (e.g., pneumonia, sepsis) • Edema • Diffuse alveolar hemorrhage • EVALI or other toxic inhalation • Connective tissue disease-associated lung disease (SLE, dermatomyositis) • Noninfectious sequelae of HSCT (e.g., PERDS, IPS) • Acute eosinophilic pneumonia • TRALI
Hypersensitivity pneumonitis	• Poorly defined centrilobular nodules • Ground-glass opacity • Lobular air-trapping	• Hypersensitivity pneumonitis (e.g., reaction to avian antigen, fungi) • EVALI
Eosinophilic lung disease		
Simple pulmonary eosinophilia	• Migratory, nonsegmental, sometimes nodular consolidation and/or ground-glass opacity	• Infection (particularly fungal and parasitic) • Organizing pneumonia • Vasculitis
Acute eosinophilic pneumonia	• Ground-glass opacity • Bronchovascular bundle and interlobular septal thickening • Pleural effusions	• Edema • Smoking (first-time, increase in amount, resumption after abstinence) • EVALI
Chronic eosinophilic pneumonia	• Peripheral predominant consolidation and ground-glass opacity	• Organizing pneumonia • Vasculitis
Capillary leak	• Interlobular septal thickening • Ground-glass opacity • Pleural effusions +/- pericardial effusion and ascites	• Edema • PERDS following HSCT • Differentiation syndrome associated with leukemia treatment • Hemophagocytic lymphohistiocytosis • COVID-19 associated MIS-C

*EVALI,* electronic cigarette or vaping product use-associated lung injury; *GVHD,* graft-versus-host disease; *PTLD,* post-transplant lymphoproliferativedisorder; *SFTPC,* surfactant protein C; *COPA,* coatomer protein complex subunit alpha; *SAVI,* STING-associated vasculopathy with onset in infancy; *SLE,*systemic lupus erythematosus; *HSCT,* hematopoietic stem cell transplant; *PERDS,* peri-engraftment respiratory distress syndrome; *IPS,* idiopathic pneumonia syndrome; *TRALI,* transfusion-related acute lung injury; *MIS-C,* multisystem inflammatory syndrome in children

### Organizing pneumonia

**Fig. 6:**
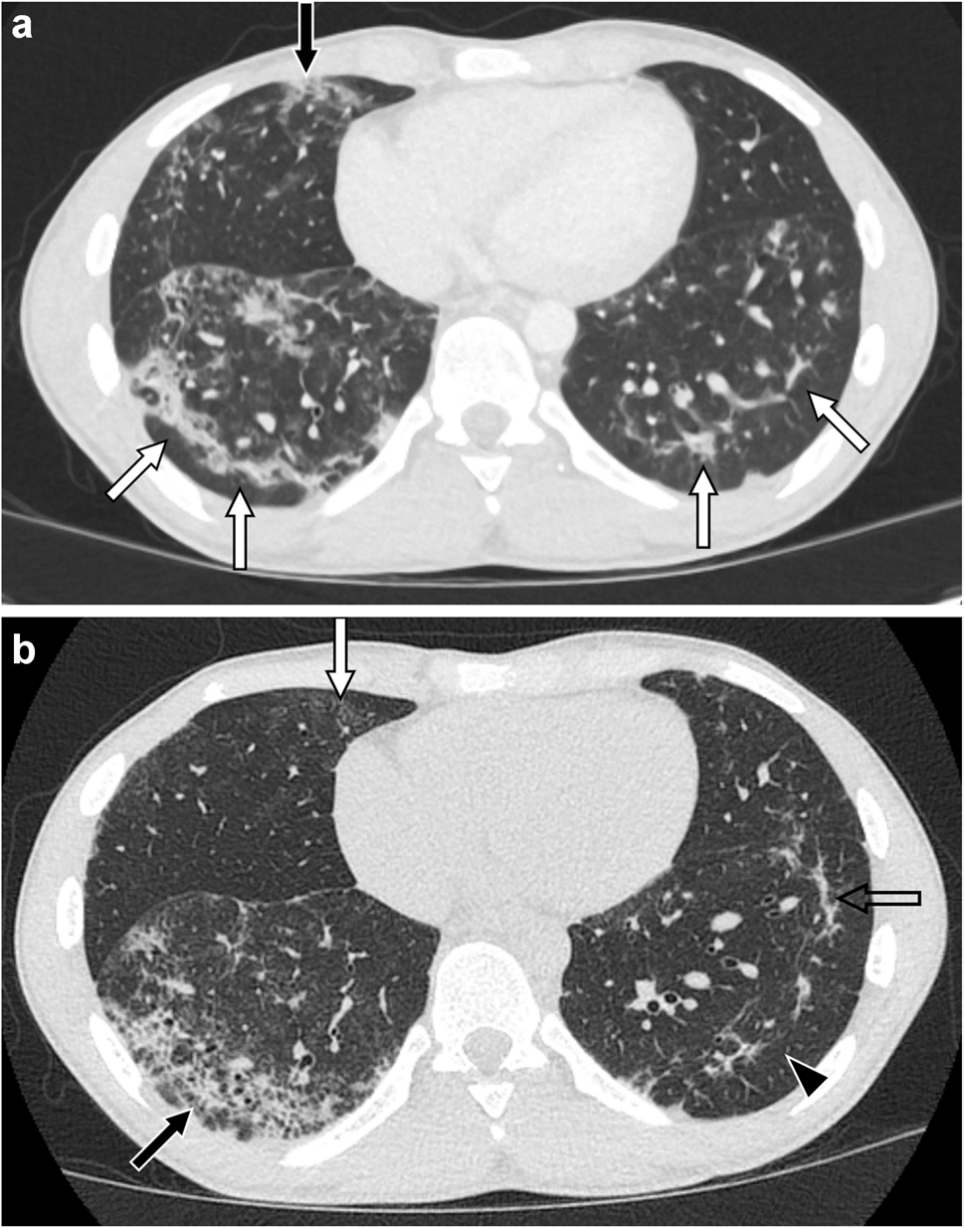
An 18–year-old male who developed dyspnea and dry cough over the course of several weeks while receiving chemotherapy that included bleomycin for testicular mixed germ cell tumor. He had a history of cigarette smoking and vaping nicotine, but quit during treatment, and did not exhibit clinical evidence of infection at the time of evaluation for his respiratory symptoms. **a** Axial CT image with contrast showing peripheral consolidation in the right middle lobe (*black arrow*) and bilateral peripheral perilobular opacities (*white arrows*) typical of organizing pneumonia. **b** Axial CT without contrast obtained 2 weeks later showing peripheral consolidation and reticulation in the right lower lobe associated with bronchial/bronchiolar dilation (*black arrow*), mild ground-glass opacity remaining at prior site of right middle lobe consolidation (*white arrow*), and areas of decreased (*arrowhead*) and increased (*open arrow*) perilobular opacity in the left lower lobe. A presumptive diagnosis of bleomycin-induced lung disease was made based on clinical presentation and imaging findings. His symptoms improved upon completion of chemotherapy and initiation of steroids, further supporting the presumptive diagnosis

**Fig. 7:**
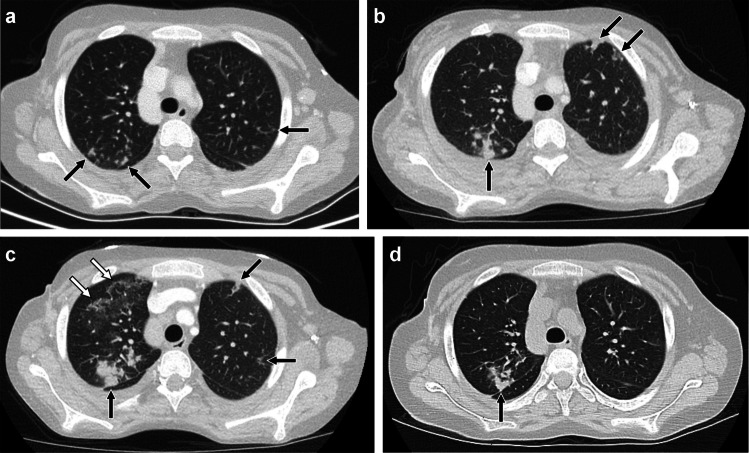
A 17-year-old female with epidermolysis bullosa who developed asymptomatic waxing and waning opacities on chest CT while receiving cemiplimab (an immune checkpoint inhibitor targeting programmed death receptor-1) for squamous cell carcinoma. Bronchoscopy with bronchoalveolar lavage and transbronchial biopsy demonstrated increased macrophages without evidence of infection, malignancy, or vasculitis. **a-c** Axial images from three chest CTs with contrast obtained over the course of 4 months showing waxing and waning/migratory upper lobe peripheral predominant nodular foci of consolidation (*black arrows*) as well as perilobular and ground-glass opacities (*white arrows*) suggesting an organizing pneumonia pattern. **d** Axial CT image without contrast obtained 1 month later, after a course of steroids for suspected immune checkpoint inhibitor-induced lung disease, showing improvement in bilateral upper lobe findings from the most recent prior chest CT, with decreased nodular consolidation remaining in the right upper lobe (*black arrow*)

OP typically manifests as peribronchovascular and/or peripheral areas of consolidation with or without ground-glass opacity. However, OP can also manifest as perilobular opacities seen as curvilinear arcadelike or polygonal opacities thicker and less sharply defined than thickened interlobular septa, the “reversed halo” or “atoll” sign in which focal ground-glass opacity is surrounded by crescentic or ring-shaped consolidation, or nodules that tend to be peribronchial/peribronchiolar with irregular or ill-defined margins and with air bronchograms when larger [51–55] (Figs. 6 and 7). Imaging findings of OP can be migratory [56, 57]. Differential diagnosis for this pattern is broad and includes infection, connective tissue disease, vasculitis, inhalation injury such as aspiration and electronic cigarette or vaping product use-associated lung injury (EVALI), radiation therapy, noninfectious sequelae of transplantation such as graft-versus-host disease and post-transplant lymphoproliferative disorder, and eosinophilic pneumonia [53, 58–63].

### Nonspecific interstitial pneumonia

NSIP tends to present as bilateral, symmetric, lower lobe predominant ground-glass opacity, reticulation, and traction bronchiectasis/bronchiolectasis with a peribronchovascular and/or peripheral axial distribution [64, 65]. NSIP is often associated with connective tissue diseases, but rarely may be familial or idiopathic [64, 66]. Surfactant dysfunction mutations (e.g., SFTPC mutation) and the type 1 interferonopathies coatomer protein complex subunit alpha (COPA) syndrome and STING-associated vasculitis with onset in infancy (SAVI) are pediatric conditions that can also present with ground-glass opacity, reticulation, and traction bronchiectasis/bronchiolectasis. However, these conditions do not tend to have the basilar predominant distribution of disease classically associated with NSIP, they all tend to have associated cysts and COPA syndrome tends to have associated nodules, and the clinical presentation is often quite distinct [50, 67–70].

### Diffuse alveolar damage

**Fig. 8:**
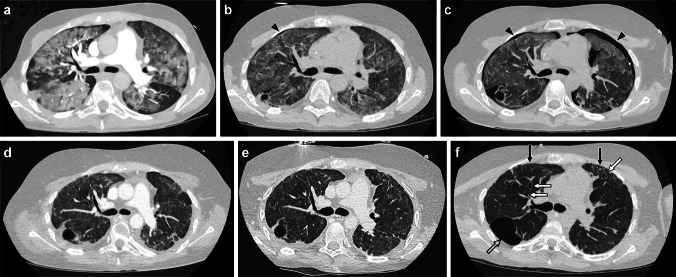
A 19-year-old male with history of acute myeloid leukemia and multiple hematopoietic stem cell transplants, most recently 18 months prior, who received cefepime and vancomycin for leukocytosis identified during work-up of poor weight gain, and who upon completion of his cefepime course developed acute respiratory failure. **a** Axial CT image with contrast showing extensive bilateral consolidation and ground-glass opacity typical of diffuse alveolar damage. **b-f** Axial CT images from five chest CT exams obtained during the subsequent year showing evolution of diffuse alveolar damage with initial replacement of consolidation by ground-glass opacity and reticulation, subsequent resolution of ground-glass opacity and decrease in reticulation, and ultimately stable reticulation (*black arrows*) and bronchiectasis/bronchiolectasis (*white arrows*) compatible with fibrosis. Pneumothoraces were also transiently seen (*arrowheads*) and a right lower lobe pneumatocele developed (*open arrow*) during the clinical course. After extensive work-up, a diagnosis of medication-induced lung disease was thought most likely and steroids, abatacept, and infliximab were administered during the clinical course with clinical improvement noted

Findings of DAD depend on the timing from initial lung insult, with early findings reflecting the intra-alveolar and alveolar wall edema as well as hyaline membranes predominating in the first week (acute or exudative phase), later findings reflecting the cellular proliferation (e.g., type 2 pneumocytes and fibroblasts) and fibrosis that predominate beyond the third week (organizing or proliferative phase), and with a mixture of these findings seen from week one to three [71] (Fig. 8). The acute/exudative phase is characterized by extensive ground-glass opacity, often along with interlobular septal thickening, areas of consolidation, and dependent atelectasis. In the organizing/proliferative phase, a greater degree of dependent-predominant consolidation and lung volume loss is seen, along with the development of bronchiectasis/bronchiolectasis that may or may not resolve at follow-up [53]. At long-term follow-up, fibrosis manifesting as reticulation, parenchymal bands, and traction bronchiectasis/bronchiolectasis often remains. Aside from medication-induced lung disease, a wide range of both direct pulmonary insults (e.g., infection, aspiration, and EVALI) and systemic processes (e.g., sepsis, pancreatitis, connective tissue disease, and noninfectious sequelae of hematopoietic stem cell transplantation such as idiopathic pneumonia syndrome and TRALI) can result in a DAD pattern of lung injury [40, 59, 72–76]. Pulmonary edema could also mimic diffuse alveolar damage acutely but should resolve relatively quickly and associated effusions are more likely to be present.

### Hypersensitivity pneumonitis

The classic features of HP include poorly defined centrilobular nodules, ground-glass opacity, and lobular air-trapping, although not all of these findings are always present [77, 78]. Although this pattern is classically associated with exposure to allergens from birds or fungi, EVALI has emerged in recent years as another potential etiology for this pattern [59, 79, 80]. Recently, it has been proposed that this pattern of lung disease be termed bronchiolocentric interstitial pneumonia, with the term hypersensitivity pneumonitis reserved for the multidisciplinary diagnosis of hypersensitivity pneumonitis, given that etiologies other than hypersensitivity pneumonitis can account for the airway-centric findings seen at imaging and histology [64].

### Simple pulmonary eosinophilia and eosinophilic pneumonia

**Fig. 9:**
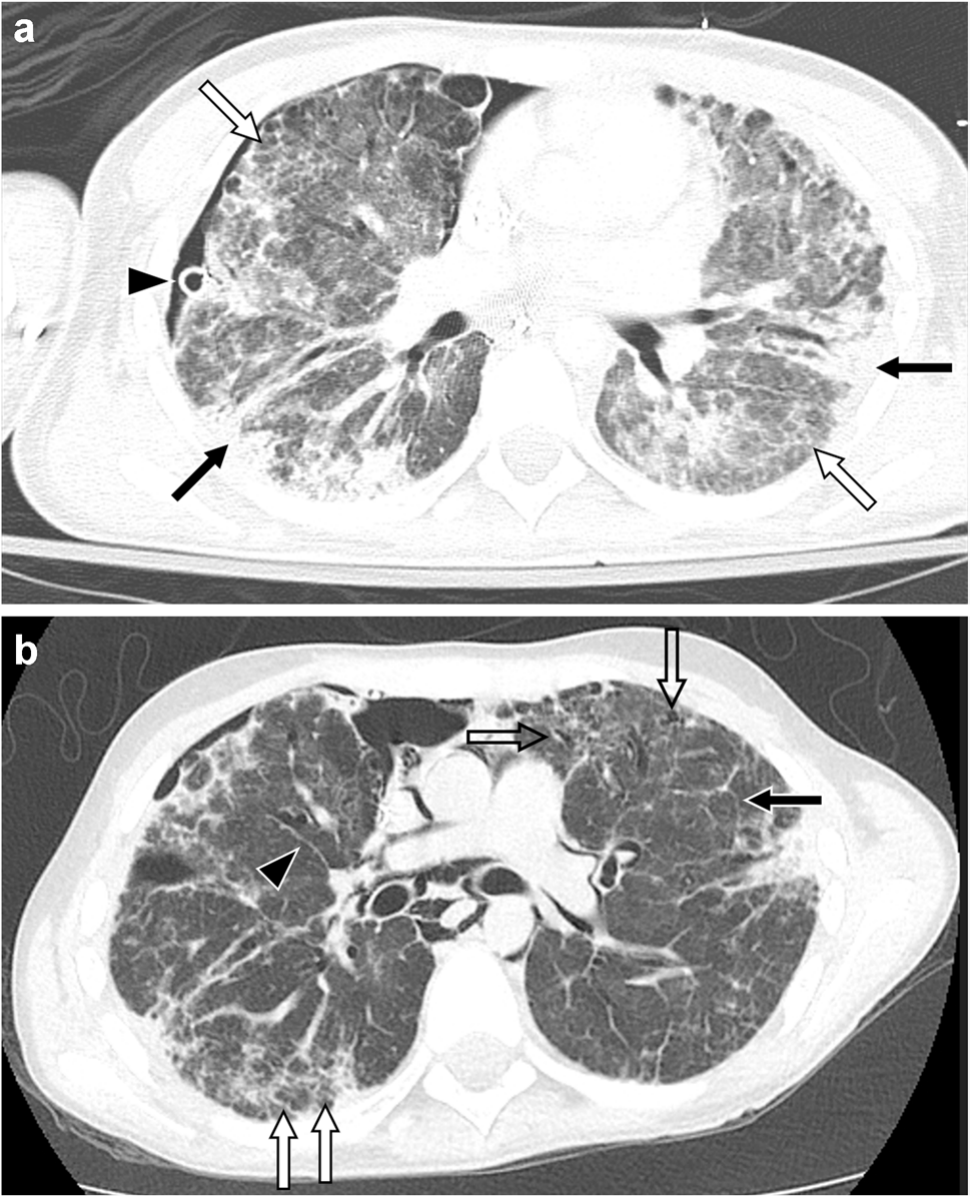
A 14-year-old receiving trimethoprim-sulfamethoxazole for acne who developed acute respiratory distress syndrome related to acute eosinophilic pneumonia. **a** Axial CT image without contrast showing diffuse bilateral ground-glass opacity, multifocal bilateral consolidation (*black arrows*) reticulation (*white arrows*), and a small right pneumothorax with chest tube in place (*arrowhead*). **b** Axial image from follow-up CT without contrast obtained 7 weeks later after interval corticosteroid treatment and discontinuation of the trimethoprim-sulfamethoxazole showing decreased ground-glass opacity and consolidation, consistent with resolving medication-induced lung disease. Interlobular septal thickening (*black arrow*), reticulation (*white arrows*), and band-like opacities (*arrowhead*) associated with bronchiolectasis (*open arrows*) remain suggesting residual fibrosis

**Fig. 10:**
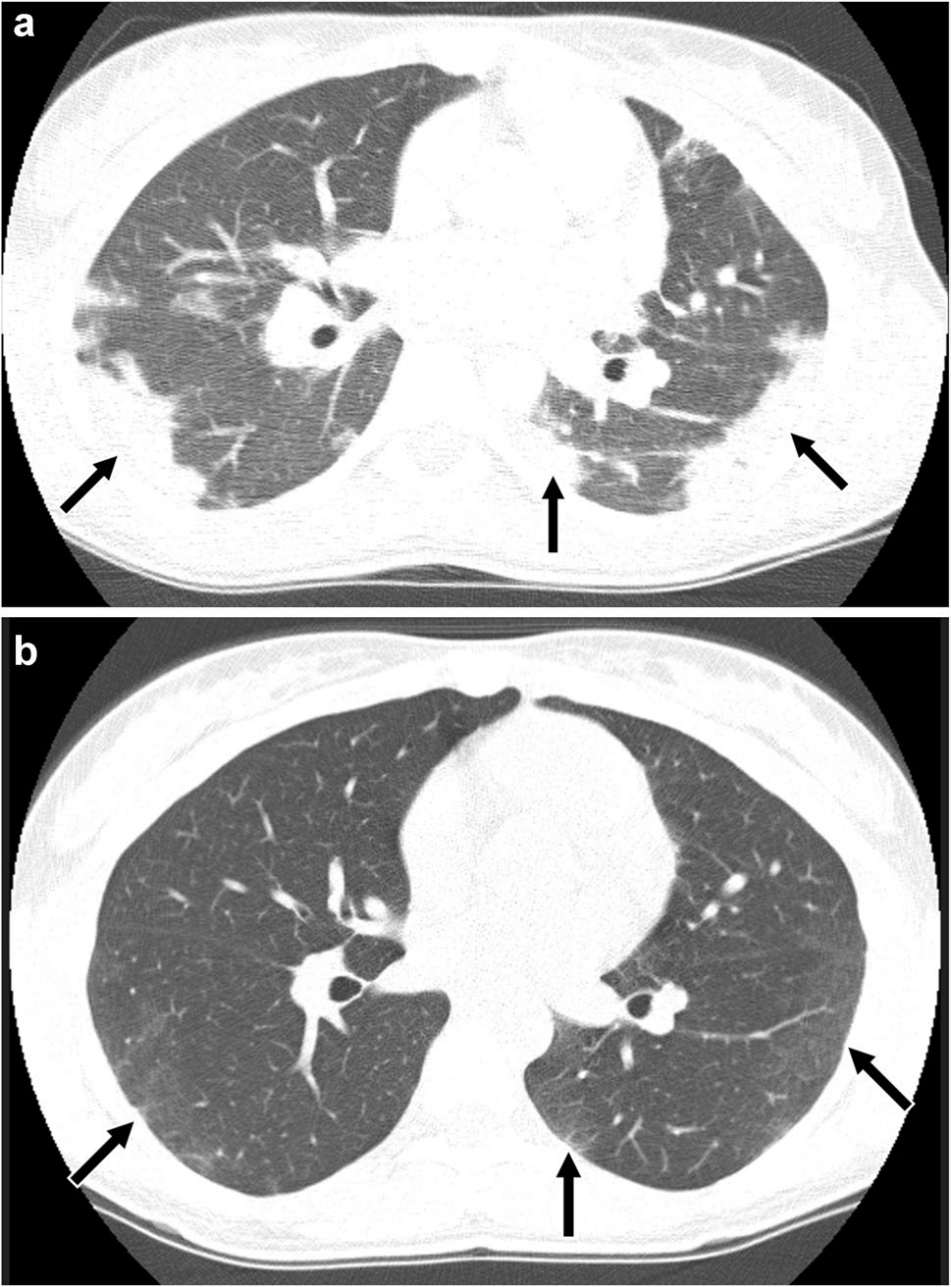
An 18-year-old female with a 3-week history of fever, cough, and dyspnea while receiving minocycline for acne. **a** Axial CT image with contrast showing bilateral peripheral consolidation (*black arrows*) typical of chronic eosinophilic pneumonia. **b** Axial image from follow-up CT with contrast obtained after medication discontinuation and 2 weeks of steroid therapy showing substantial improvement, with only mild patchy peripheral ground-glass opacity (*black arrows*) remaining at prior sites of consolidation consistent with minocycline-induced lung disease. (Images reprinted from Caffey’s Pediatric Diagnostic Imaging, 12th Edition, Zucker EJ, Guillerman RP, Fishman MP, Casey AM, Lillehie CW, Lee EY, Diffuse Lung Disease, 594–604, 2013, with permission from Elsevier.)

Simple pulmonary eosinophilia is characterized by peripheral eosinophilia and minimal or no pulmonary symptoms, and manifests on CT as transient and migratory, single or multiple nonsegmental areas of consolidation or ground-glass opacity, sometimes nodular in appearance, that resolve spontaneously within one month, a pattern that could be seen with certain infections including fungal and parasitic, organizing pneumonia, or vasculitis [81, 82]. Acute eosinophilic pneumonia tends to manifest as bilateral areas of ground-glass opacity, interlobular septal thickening, bronchovascular bundle thickening, and pleural effusions similar to pulmonary edema, with fever and absence of cardiomegaly helpful clues to the diagnosis of the former [83–85]. Areas of consolidation are also often seen [83, 85, 86] (Fig. 9). Notably, peripheral eosinophilia may be absent at presentation in acute eosinophilic pneumonia [87]. Causes of acute eosinophilic pneumonia in children aside from medication-induced lung disease include new-onset or increased smoking as well as EVALI [59, 80, 86, 87]. In the setting of chronic eosinophilic pneumonia, peripheral predominant areas of consolidation and ground-glass opacity are most frequently seen, with perilobular opacities or the “reversed halo” sign also sometimes present, all findings resembling organizing pneumonia except that associated peripheral eosinophilia is usually present [83, 88] (Fig. 10). Primary differential diagnosis for this pattern of eosinophilic lung disease includes organizing pneumonia and vasculitis.

### Capillary leak

**Fig. 11:**
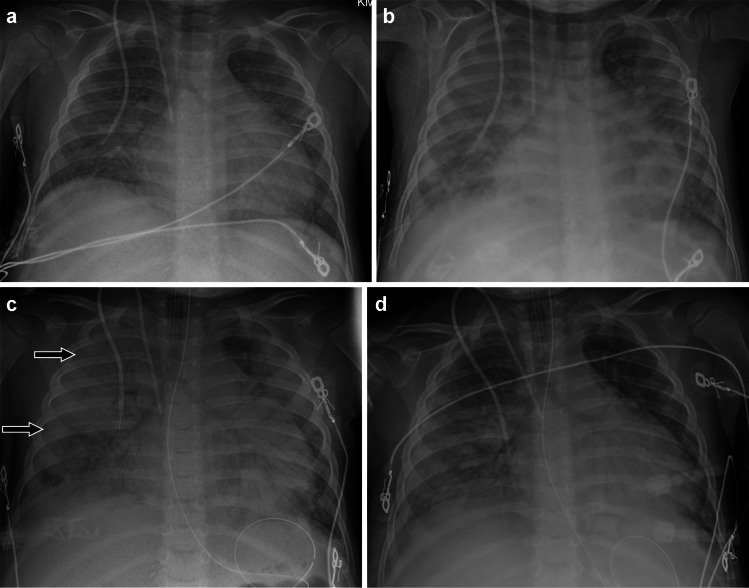
A 3-year-old male with neuroblastoma who developed acute bronchospasm during dinutuximab (a monoclonal antibody targeting glycolipid GD2) infusion and subsequent respiratory failure requiring intubation in the setting of clinical evidence of capillary leak. Infection work-up was negative. **a-d** Anteroposterior chest radiographs obtained over the course of 4 days showing rapid development of widespread bilateral consolidation and a right pleural effusion (*black arrows*), and subsequent rapid decrease in findings. The temporal relationship of the respiratory condition to dinutuximab infusion and the imaging findings support the diagnosis of dinutuximab-induced capillary leak syndrome, an adverse effect known to occur with dinutuximab

Capillary leak appears as diffuse interlobular septal thickening and ground-glass opacity with or without consolidation, as well as pleural effusions indistinguishable from pulmonary edema [89] (Fig. 11). Pericardial effusion and ascites may also be visible. Other than cardiogenic pulmonary edema, differential diagnosis considerations include hyperinflammatory conditions often associated with capillary leak including peri-engraftment respiratory distress syndrome (PERDS) following hematopoietic stem cell transplant, differentiation syndrome associated with leukemia treatment, hemophagocytic lymphohistiocytosis (HLH), and multisystem inflammatory syndrome in children (MIS-C) associated with COVID-19 [63, 90–95].

Although the differential diagnosis for these lung disease patterns can be broad, many of the possibilities can be excluded by review of a given patient’s clinical history and presentation, and the remaining considerations can often be distinguished through further clinical evaluation. A limited actionable differential diagnosis, in which correlation with clinical presentation and further diagnostic testing by the clinical team can help distinguish between differential diagnosis possibilities, should generally be the goal when interpreting chest CT, and in our experience is often possible. Even suggesting the possibility of medication-induced lung disease, when appropriate, can be helpful, as it may not yet have been considered.

## Conclusion

Establishing the diagnosis of medication-induced lung disease in children is challenging, but the pediatric radiologist may be the first to consider the diagnosis. Knowledge of the classes of medications that tend to be associated with medication-induced lung disease and the clinical scenarios in which those medications are used as well as the recognizable patterns of medication-induced lung disease on CT can help the pediatric radiologist identify when to include this entity in the differential diagnosis. Key take-home points are as follows:

- Consider medication-induced lung disease in any patient receiving chemotherapy, an immune checkpoint inhibitor, a molecular targeting agent, a DMARD, and/or an antibiotic with otherwise unexplained pulmonary findings on imaging.
- Consider medication-induced lung disease if any of the above-described CT patterns of lung disease are identified, particularly if the clinical scenario is one in which medication-induced lung disease is more commonly seen and/or if a potential causative medication can be identified in the patient’s medication list.
- Medication-induced lung disease is essentially a diagnosis of exclusion, and the differential diagnosis can be broad, but recognizing certain patterns of lung disease at CT helps narrow the differential diagnosis.
- Although even just the suggestion of medication-induced lung disease in the radiology report can be helpful, the diagnosis of medication-induced lung disease is ultimately best established through multidisciplinary collaboration including the radiologist.

## Data Availability

No datasets were generated or analysed during the current study.
